# Research on the effect of uncertain rewards on impulsive purchase intention of blind box products

**DOI:** 10.3389/fnbeh.2022.946337

**Published:** 2022-08-15

**Authors:** Yi Zhang, Hang Zhou, Jian Qin

**Affiliations:** School of Economics and Management, Shanghai Institute of Technology, Shanghai, China

**Keywords:** blind box, perceived uncertainty, impulsive purchase intention, perceived luck, curiosity

## Abstract

Since 2019, China has gradually seen a “blind box” boom, and young people have quickly become the main buying force of blind boxes, promoting the continuous development of the blind box industry. Previous studies have shown that uncertainty in events with positive prospects can play a more positive role than certainty. However, how does uncertainty in the blind box affect consumers’ emotions and cognition and trigger subsequent consumption decisions? To clarify the internal mechanism of this process, this paper takes the blind box as the research object and constructs the mechanism model of perceived uncertainty on consumers’ impulsive purchase intention, based on Stimulus-Organism-Response (SOR) theory. In addition, the curiosity variable and perceived luck variable are introduced according to the information gap theory and optimism theory. On this basis, we conduct an empirical analysis by means of a questionnaire survey. The results show that perceived uncertainty has a positive impact on consumers’ impulsive purchase intentions, in which curiosity plays a mediating role. Besides, perceived luck positively moderates the impact of perceived uncertainty on impulsive purchase intention. This study clarifies the internal impact of perceived uncertainty on impulsive purchase intention of the blind box and enriches the basic theory of uncertainty reward and purchase intention. At the same time, we also offer related recommendations for future enterprises to learn from the marketing model of uncertain rewards.

## Introduction

Blind boxes refer to opaque boxes containing a wide variety of fashionable toys, which since their release, have been sought after by many consumers as soon as it comes out. The highlight of blind box marketing lies in its inherent “blind” characteristic. Drawing a blind box is equivalent to drawing an indefinite reward with great randomness. Nowadays, many young people are yearning for interesting consumption experiences, and products such as blind boxes meet these needs. According to data, more than 200,000 consumers spent tens of thousands of yuan on blind boxes in 2020, of which the post-1995 generation comprised the largest share. The size of the domestic tide play market has increased from 6.3 billion yuan in 2015 to 29.48 billion yuan in 2020 and is expected to exceed 150 billion yuan by 2025. Although the marketing model of blind boxes has achieved an excellent response, how does the uncertain reward attract consumers? What are the consumers’ cognitions and emotions behind this phenomenon? For relevant enterprises, managers need to deeply understand consumers’ emotional needs in terms of uncertain incentive, and formulate various strategies to improve consumers’ brand attitudes and the possibility of consumption, which is of great significance for enterprises to expand brand influence and maintain brand vitality.

Previous research in psychology and economics generally understands uncertainty to be negative, which will reduce people’s expected utility and lead to pain and anxiety. People usually prefer certainty and avoid situations with uncertain results (Calvo and Dolores Castillo, 2001). However, many studies in the field of marketing have pointed out that uncertainty is not entirely negative. On the contrary, sometimes a certain degree of uncertainty can stimulate people’s emotions and motivate follow-up actions. In the consumption experience, an uncertain reward can stimulate consumers’ willingness and behavior more than a definite reward (Zhang et al., 2017). Moreover, when consumers’ decisions are based on emotions, the possibility of purchase caused by the uncertain reward will increase (Laran and Tsiros, 2013). Although existing studies have confirmed that uncertain rewards can predict the improvement of marketing performance, some studies ignore consumers’ cognitive and conscious processes. Furthermore, while some scholars have explored this internal mechanism, this is mainly based on the perspective of positive emotional experience. Therefore, it is still necessary to further explore the boundary conditions of the impact of reward uncertainty on motivation.

At present, the research on blind box consumption is still in its infancy. Many scholars have widely discussed the consumer market and consumer psychology under the blind box economy, but most studies are based on analyses of phenomena or theories, and there is still a lack of systematic empirical testing. Some existing empirical studies mainly explore the internal mechanism of blind box purchases from the perspective of consumption experience and consumption motivation (Yan and Wu, 2021; Wang et al., 2022). However, no scholars have explored the impact of perceived uncertainty on impulse consumption from the perspective of reward uncertainty. Therefore, based on previous studies, this paper takes the blind box as the research object to further explore the internal relationship between perceived uncertainty and impulsive purchase intention, and explore whether curiosity and perceived luck play important roles in it. Not only does this study promote theoretical research on uncertain rewards, it also deepens theoretical research of blind box marketing, which provides a certain theoretical basis and decision-making basis for subsequent related enterprises to carry out uncertain reward marketing activities.

## Theoretical background

### Uncertain reward

Perceived uncertainty is a sense of the unknown that people experience when faced with future events involving many possible outcomes (Quintal et al., 2010). The traditional economic theory believes that consumers will avoid the uncertainty in income selection (Von Neumann and Morgenstern, 2007). Therefore, many enterprises in the past committed to taking various measures to reduce the uncertainty consumers face, and provide consumers with certain products and services. However, recent studies have shown that uncertainty is not always negative. In fact, high uncertainty can activate the brain’s reward system and enhance an individual’s learning ability and behavioral motivation (Jezzini et al., 2021). Moreover, the uncertain reward is also associated with hedonic ability and the decision to take risks (Pizzagalli et al., 2008; Guo et al., 2013).

There is also a large body of psychology and marketing research that demonstrates the positive effect of uncertain rewards. Lee and Qiu (2009) explored the influence of uncertain rewards in events with positive prospects on consumer sentiment, that is, individuals know that the future outcome of the event is positive, but do not know which outcome to achieve. They found that uncertain rewards could bring individuals more lasting emotional experiences than certain rewards. Laran and Tsiros (2013) explored the promotional effect of uncertainty on freebies under different consumption decisions, and the results showed that if consumers made decisions based on emotion, uncertain rewards would bring them surprise and pleasure, which will promote the improvement of marketing performance. Shen et al. (2015) investigated the possible positive effects and applicable conditions of uncertain rewards. It was found that when people pay more attention to the process of pursuing uncertain rewards rather than the results, they will get excited. In addition, uncertain rewards play a great incentive role, increasing the resource investment in the process of pursuing rewards. Shi et al. (2021) studied the relationship between uncertain reward and donation intention, and pointed out that an uncertain reward would lead to lower psychological pain of donation. People’s donation intentions would be higher after receiving uncertain rewards rather than specific rewards. Shou et al. (2021) discussed the remedial effect of betting games based on uncertain settings on service failure, and the research showed that betting games based on uncertain settings can effectively improve customers’ satisfaction. Zhang et al. (2021) studied the essence of freebies promotion and found that giving freebies can significantly predict customer delight more than not giving freebies, in which perceived uncertainty plays a significant moderating role.

To sum up, previous studies have shown that consumers prefer uncertain rewards to certain rewards in certain situations. In marketing activities, the reasonable use of uncertainty can induce the emotional needs of consumers and promote sales growth. However, most studies on the influence of uncertain rewards on consumption decisions are based on the emotional perspectives of surprise, pleasure, and so on. Whether this process is also affected by other factors has yet to be explored.

### Information gap theory

The information gap is the gap between what individuals already know and what they want to get. When people realize that they have an information gap, they will be curious, which will further motivate them to take action to fill the information gap (Loewenstein, 1994). When faced with a huge information gap, people will pay attention to the information they know, but as the gap gradually narrows, the focus will turn to the information gap, thereby strengthening individual curiosity.

Menon and Soman (2002) found that advertising strategies that stimulate curiosity can improve a user’ interest and learning ability. Compared with obtaining complete advertising information, limited advertising information will encourage consumers to actively search for more information. Brod and Breitwieser (2019) pointed out that curiosity can be stimulated by predicting whether one’s inner desire will be satisfied. When participants generate predictions, the personal information gap will also increase in the process of expecting the correct answer, thus further reinforcing curiosity. Singh and Manjaly (2021) designed an experiment on a missing letter to test participants’ curiosity. The second, fourth, and seventh letters of a word were missing, and each participant was required to complete them. The results showed that both the information gap (missing letters) and the participants’ uncertainty about the whole word predicted their curiosity about learning the whole word. When the information gap is bridged, participants will feel satisfied.

Overall, the information gap theory focuses on explaining people’s attention to the process of pursuing uncertain rewards. When people receive information, if they first experience incomplete information instead of directly receiving the determined information, they will have a better overall experience, which is conducive to stimulating more positive attitudes or more favorable behavioral tendencies (Ruan et al., 2018). Based on the information gap theory, this study explores the internal influence of perceived uncertainty on consumers’ impulsive purchase intentions.

### Optimism theory

Optimism is an individual difference variable that reflects people’s generally favorable expectations for the future (Carver et al., 2010). According to the innate optimism theory, individuals will maintain an optimistic attitude under uncertain circumstances and subconsciously consider themselves lucky, which is manifested by exaggerating the probability of expected events or decreasing the probability of unexpected events (Taylor and Brown, 1988; Bar-Hillel and Budescu, 1995).

Many studies show that an individual’s optimism has an important impact on their consumption intention and behavior. Lee and Qiu (2009) pointed out that in uncertain marketing activities, consumers may mentally imagine favorable prospects for them. That is, a psychological intention can occur even without the real sensory or perceptual experience of real objects, enabling consumers to experience more lasting positive emotions. Goldsmith and Amir (2010) explored the boundary conditions of uncertain promotions through experiments. They found that when individuals’ innate optimism was impaired, uncertainty motivation did not lead to positive outcomes, suggesting that an individual’s innate optimism response is a valid cause of uncertainty motivation. Ailawadi et al. (2014) studied the effectiveness of uncertain discount promotion and found that consumers tend to estimate the probability of occurrence of favorable outcomes higher. Hock et al. (2020) explores how promotional games increase consumer conversion and spending. Hock found that winning in the promotional game would affect the individual’s positive attitude toward the store and optimistic tendency toward the future, and these factors together lead to higher purchase possibility and average overall expenditure.

In a word, optimism theory focuses on explaining people’s attention to the outcome of pursuing uncertain rewards. In uncertain situations, individuals’ positive assumptions of their own luck can strengthen achievement motivation, and then make corresponding behavioral responses with the subjective perception of whether they are lucky or not. Based on optimism theory, this study explores the internal influence of perceived uncertainty on consumers’ impulsive purchase intentions.

### Stimulus-organism-response theory

Stimulus-Organism-Response (SOR) theory was developed by Mehrabian and Russell (1974). It explains the influence of external stimuli on individual cognition, emotion, and behavior. Since then, many scholars have applied this theory to the psychology and consumer behavior research fields.

Each series of blind boxes consists of 12 regular items and 1 hidden item, and only when you open the box can you know which one is selected. Therefore, we regard this unknown reward mechanism as an external stimulus. Previous studies on external stimuli based on SOR theory were mostly measured from the perspective of individual perception, such as perceived information quality and perceived interaction (Thomas and Mathew, 2018; Zhu et al., 2020). Therefore, this study adopted the variable of perceived uncertainty to measure external stimuli. Scholars usually regard the “O” in the model as individual cognition and emotion, such as perceived trust and perceived entertainment (Huang et al., 2020). Therefore, this study takes the consumers’ curiosity as an organism factor. Moreover, “R” in the model represents the individual response based on cognition and emotion. In previous studies, purchase intention was often considered to be a response factor, so this study takes impulsive purchase intention as an individual response. It follows that this paper studies the relationships among perceived uncertainty, curiosity, and impulse purchase intention based on the SOR theoretical model.

## Research hypotheses and conceptual model

### Perceived uncertainty and impulse purchase intention

Perceived uncertainty refers to an individual’s lack of understanding due to unknown or unreliable information (Becker and Knudsen, 2005). Earlier, Holbrook (1986) pointed out that shopping value is closely related to an impulsive purchase. As one of the purchase values, hedonic shopping value can be understood as the joy, enjoyment, and other emotional pleasures experienced by consumers in the shopping process, which can strengthen unplanned purchases (Wood, 2005). In the same way, uncertain rewards in the process of consumption experience can bring consumers a sense of surprise and stimulate subsequent purchase decisions (Laran and Tsiros, 2013). Furthermore, Yang et al. (2017) argued that uncertain rewards can slow down consumers’ hedonic adaptation. As time goes on, repeated deterministic consumption experiences will make consumers feel dull and will not increase happiness. Nevertheless, if consumers have the opportunity to get rewards, they will be full of expectations for rewards and even be willing to take risks to get the rewards. Therefore, it can be argued that uncertain products such as blind boxes can, to some extent, alleviate consumers’ hedonic adaptation and trigger impulse consumption.

Based on the above theories, the following hypothesis is proposed:

*H1- Perceived uncertainty has a positive impact on impulse purchase intention.*

### The mediating role of curiosity

Curiosity is usually defined as an individual’s psychological desire to explore novelty and the unknown (Berlyne, 1978). According to the information gap theory, information gaps occur in uncertain situations, in which an individual’s curiosity is aroused and they will take further actions to meet their cognitive needs (Loewenstein, 1994). In addition, van Lieshout et al. (2021) studied the main effect of an uncertain outcome on curiosity intensity. It was found that curiosity increased as the uncertainty of the outcome increased, regardless of whether the final result was profitable or not. In brief, curiosity reflects the drive to reduce unpleasant uncertainty.

Based on the above theories, the following hypothesis is proposed:

*H2a- Perceived uncertainty has a positive impact on curiosity.*

Emotion is an important source of information. A person’s emotional state will affect their information processing and judgment and even affect their willingness and action independently of their self-cognition (Bagozzi et al., 1999). In terms of marketing communication, consumers’ moods and feelings will have an important impact on their purchase intention behaviors, and there is a correlation between unplanned purchases and consumers’ emotions (Kaltcheva and Weitz, 2006). Curiosity, as the most basic and universal emotion of individuals, reflects an individual’s desire for unknown information, which has a significant incentive component to stimulate interest or eliminate uncertainty, thus driving people to engage in irrational exploration behaviors (Gruber and Ranganath, 2019). Hill et al. (2016) also investigated the role of curiosity in influencing consumer behavior and found that purchase motivation would correlate positively with the improvement of curiosity.

Based on the above theories, the following hypothesis is proposed:

*H2b- Curiosity has a positive impact on impulse purchase intention.*

### The moderating role of perceived luck

Perceived luck reflects an individual’s attitude toward his luck, which plays a special role in guiding individuals to pay more attention to the positive aspects of events (Wohl and Enzle, 2003; Day and Maltby, 2005). In general, if people cannot reasonably attribute things within their cognitive scope, they will ascribe the result to luck. Individuals with good luck beliefs are usually self-centered and show strong positive cognitive bias. Even in uncontrollable or high-risk situations, individuals with high perceived luck can keep looking forward to unknown or future things and are more daring to take risky or impulsive behaviors (Yarritu et al., 2014). Given this personality attribute, we can see that many enterprises have designed corresponding marketing strategies, such as lotteries, promotional activities, and so on (Ward and Hill, 1991). In this case, an individual’s belief in good luck can be used as an optimistic coping strategy. That is to say, positive assumptions about luck can bring people a sense of confidence and control, making them spend more in subsequent purchasing activities (Ward and Hill, 1991; Darke and Freedman, 1997a).

Based on the above theories and the content of Hypothesis 1, the following hypothesis is proposed:

*H3- Perceived luck positively moderates the impact of perceived uncertainty on impulsive purchase intention.*

### Construction of theoretical model

**FIGURE 1:**
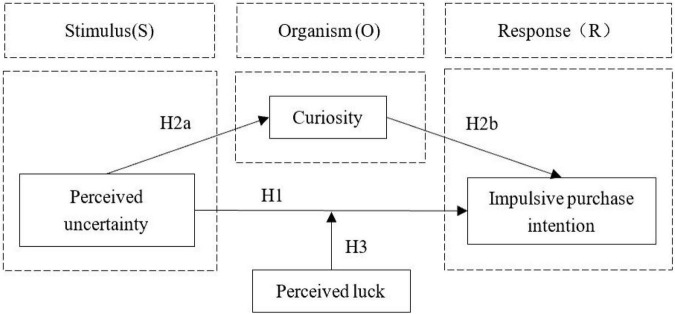
Conceptual model.

Based on the above theoretical analysis and research hypotheses, a theoretical model of the impact of perceived uncertainty on consumers’ impulsive purchase intention is constructed (shown in Figure 1).

## Study design and data processing

### Questionnaire design

The first part of the questionnaire is about the scale items of the four variables, including perceived uncertainty, impulsive purchase intention, curiosity, and perceived luck. In order to ensure the content validity of the scale, the mature scale was used to design relevant items. Perceived uncertainty refers to the perceived uncertainty scale (3 items) revised by Zhang and Liu (2010). Impulsive purchase intention refers to the impulsive purchase intention scale (3 items) revised by Zhang and Kong (2021). Curiosity refers to the curiosity scale (4 items) developed by Kashdan et al. (2009). Perceived luck refers to the belief in good luck scale (10 items) developed by Darke and Freedman (1997b). A 5-point scale was used to score all the above measurement items. The second part of the questionnaire gathers the respondents’ basic personal information.

### Reliability and validity test of pre-survey

Before the formal questionnaire is issued, 60 sample data are collected for pre-survey to test the reliability and validity of the scale design. First, we use Cronbach’s α to verify the reliability of the questionnaire. Generally, a coefficient value above 0.8 means that the questionnaire has good reliability. In the process of reliability testing, this study also tested the CITC (Corrected Item-Total Correlation) and CAID (Cronbach’s Alpha if Item Deleted) of each measurement item. As shown in Table 1, the Cronbach’s α of each variable is greater than 0.8, the CITC value of each item is greater than 0.4, and the value of the reliability coefficient after removing each item does not show a significant improvement, the scale thus passes the reliability test.

**TABLE 1:** Reliability and validity tests of the questionnaire.

Variable	Items	CITC	CAID	α	Factor loading	Cumulative variance (%)
Perceived uncertainty	PU1	0.707	0.737	0.827	0.876	74.33
	PU2	0.666	0.780		0.851	
	PU3	0.682	0.763		0.860	
Perceived luck	PL1	0.826	0.916	0.929	0.869	61.51
	PL2	0.831	0.916		0.872	
	PL3	0.843	0.915		0.883	
	PL4	0.692	0.924		0.756	
	PL5	0.694	0.924		0.753	
	PL6	0.852	0.915		0.893	
	PL7	0.815	0.917		0.864	
	PL8	0.765	0.920		0.822	
	PL9	0.447	0.932		0.513	
	PL10	0.423	0.931		0.468	
Curiosity	CT1	0.765	0.746	0.835	0.886	67.13
	CT2	0.529	0.848		0.705	
	CT3	0.685	0.783		0.838	
	CT4	0.693	0.780		0.837	
Impulsive purchase intention	PI1	0.727	0.758	0.840	0.884	75.89
	PI2	0.663	0.818		0.845	
	PI3	0.725	0.757		0.884	

In addition, we use exploratory factor analysis to test the validity of the measurement items. First, KMO and Bartlett tests are carried out for the measurement items of each variable. The results show that the KMO values of these four scales are greater than 0.7, and the significance level of the Bartlett test of each scale is 0.000, so each measurement item is suitable for factor analysis. We use principal component analysis for factor extraction and variance maximization for factor rotation to evaluate whether the measurement item is a single dimension (Weiss, 1970). As shown in Table 1, the factor load coefficients of perceived uncertainty, impulsive purchase intention and curiosity are greater than 0.6, and there are no sub-dimensions. The factor load coefficient of B9 and B10 in the perceived luck scale is less than 0.6, so it is considered to be deleted (Ma, 2002). A factor analysis was performed again after deleting these two items. The results showed that the KMO value of the perceived luck scale is greater than 0.7, and the significance level of the Bartlett test was 0.000, the percentage of explained variance increased from 61.51 to 71.42. Therefore, after small sample measurement and calculation, the measurement items of the perceived luck scale changed from 10 to 8. Combined with the survey content of this study, the structural design of the questionnaire and the expression of item semantics were further adjusted, and a formal questionnaire containing 18 items of the scale was designed.

### Data collection

The formal questionnaire was designed on the questionnaire star platform and published online. We sent the accessible links of the questionnaire to the blind box online communities of WeChat, QQ, and Xiaohongshu, and invited relevant respondents to fill it out. These groups are established for blind box communication and exchange, and include more female than male members. We also collected offline data in some blind box sales stores. Finally, we collected 219 questionnaires. By filtering out the invalid questionnaires with insufficient filling time, extreme values, and high repeatability, 193 effective questionnaires were gathered, with an effective rate of 88.13%. As shown in Table 2, males and females accounted for 28.50 and 71.50% of respondents respectively. In terms of age distribution, people aged 19–24 accounted for 53.40% of respondents, and those aged 25–30 accounted for 37.30%. In terms of educational background distribution, high school or below accounted for 6.50%, junior college accounted for 31.80%, and undergraduate and above accounted for 57.7% of respondents. In terms of occupational distribution, students accounted for 35.30% of respondents and employees of enterprises and institutions accounted for 44.80%. In terms of monthly income, 62.7% of respondents earned 6,000 yuan or less and 37.3% of respondents earned more than 6,000 yuan. In terms of purchase frequency, 58.30% of the investigated population purchased blind boxes 6 times per year or more.

**TABLE 2:** Demographic characteristics of the sample.

Classification	Characteristic index	Frequency	Percentage (%)
Gender	Male	55	28.50
	Female	138	71.50
Age	<18 years	2	1.00
	19–24 years	103	53.40
	25–30 years	72	37.30
	>31 years	16	8.30
Education	High school or below	13	6.50
	Associate degree	64	31.80
	Bachelor’s degree	95	47.30
	Master’s degree or above	21	10.40
Occupation	Students	71	35.30
	The staff of enterprises and institutions	90	44.80
	Individual freelancer	27	13.40
	Others	5	2.50
Monthly income (RMB)	<3,000	59	30.60
	3,001–6,000	62	32.10
	6,001–10,000	60	31.10
	>10,001	12	6.20
Annual purchase frequency	<2 times	10	5.00
	3–5 times	66	32.80
	6–10 times	97	48.30
	>11 times	20	10.00

## Analyses and results

### Common method biases tests

In order to avoid homologous bias, the Harman single-factor test was used to analyze the homologous bias of all valid questionnaires. Factor analysis was carried out on all the measurement items of 193 valid questionnaires. There were 4 factors with eigenvalues greater than 1 and the variance contribution rate of the first factor was 24.27%, below the critical value of 40%. Therefore, there are no significant common method biases in the survey data, and the results are trustworthy.

### Reliability and validity tests

SPSS25.0 software was used for the reliability analysis. It can be seen from Table 3 that Cronbach’s α values of all variables are greater than 0.8. To be precise, perceived uncertainty is 0.871; perceived luck is 0.899; curiosity is 0.822; impulse purchase intention is 0.870. This indicates that the reliability level of the questionnaire was acceptable, and the data analysis of the scale was reliable.

**TABLE 3:** Reliability and validity analyses of variables.

Variable	Items	Factor loading
Perceived uncertainty α = 0.871 AVE = 0.695 CR = 0.872	In the face of blind boxes, I feel unsure whether the items I draw are completely consistent with my expectations.	0.845
	In the face of blind boxes, I find it difficult to be sure whether the goods I get are suitable for me.	0.842
	In the face of blind boxes, I feel unable to judge the real material and quality level of the goods in the box.	0.813
Perceived luck α = 0.899 AVE = 0.530 CR = 0.899	Luck plays an important role in drawing a blind box.	0.639
	I consider myself a lucky person.	0.726
	I believe in luck.	0.777
	I often feel lucky when I draw a blind box.	0.791
	I always get lucky when I draw a blind box.	0.789
	Luck helps me draw the blind box I want.	0.772
	I don’t mind that drawing a blind box is like taking a chance, because I’m a lucky guy.	0.656
	I can’t decide what to draw from the blind box, but I’m lucky, so I will get what I want in the end.	0.639
Curiosity α = 0.822 AVE = 0.543 CR = 0.826	I really enjoy the uncertainty of blind boxes.	0.697
	I love the wonderful experience of seeking new things given by blind boxes.	0.701
	I prefer the exciting unpredictability of blind boxes.	0.719
	I’m the kind of person who can accept uncertainty in a blind box.	0.824
Impulsive purchase intention α = 0.870 AVE = 0.669 CR = 0.874	I see a blind box and want to buy it, even though it isn’t in my purchase plan.	0.800
	I experience a sudden desire to buy a blind box.	0.906
	I experienced a strong desire to buy a blind box that I had no intention of buying.	0.797

AMOS24.0 software was used for confirmatory factor analysis. As shown in Table 3, the AVE values of perceived uncertainty, perceived luck, curiosity, and impulse purchase intention were all greater than 0.5, and CR values were all greater than 0.8, indicating that the measurement indexes of all variables used in the questionnaire had good convergence validity. In addition, the structural validity of the model was tested. The fitting indexes were as follows: (χ^2^/df) = 1.373, SRMR = 0.041, RMSEA = 0.044, NFI = 0.911, CFI = 0.974, TLI = 0.969, IFI = 0.974, indicating that the degree of fitting of the structural model was good. The discriminative validity between variables was further analyzed. It can be seen from Table 4 that the four variables of perceived uncertainty, perceived luck, curiosity, and impulse purchase intention were significantly correlated with each other (*P* < 0.001). Moreover, the correlation coefficient values of all variables are less than their square root of AVE, indicating that the discriminant validity among variables was ideal.

**TABLE 4:** Discriminant validity test of variables.

Variable	Perceived uncertainty	Perceived luck	Curiosity	Impulse purchase intention
Perceived uncertainty	0.695			
Perceived luck	0.135***	0.530		
Curiosity	0.224***	0.206***	0.543	
Impulse purchase intention	0.127***	0.218***	0.133***	0.699
Square root of AVE	0.833	0.728	0.737	0.836

***P < 0.001. The values on the diagonal represent the AVE of each variable.

### Mediating effect test

The process plug-in in SPSS25.0 was used to analyze the mediating effect. Taking perceived uncertainty as the independent variable, impulse purchase intention as the dependent variable, curiosity as the mediating variable, and controlling the influential factors of gender, age and income, we used Model 4 in the macro program compiled by Hayes to test the mediating effect. It can be seen from Table 5 that the path coefficient of perceived uncertainty and impulsive purchase intention was significant in the direct effect model (β = 0.216, *t* = 4.583, *P* < 0.01), leading support to H1. In support of H2a, when the curiosity variable was added, the positive predictive effect of perceived uncertainty on curiosity was significant (β = 0.354, *t* = 7.378, *P* < 0.01). At the same time, curiosity could significantly positively predict impulsive purchase intention (β = 0.319, *t* = 4.708, *P* < 0.01), which provides support for H2b. In addition, the path coefficients between perceived uncertainty and impulse purchase intention were significant in both direct effect and mediating effect, but the positive predictive effect of perceived uncertainty on impulse purchase intention decreased after adding the curiosity variable (β = 0.103, *t* = 2.026, *P* < 0.05). Therefore, it could be concluded in this model, curiosity plays a partial intermediary role between perceived uncertainty and consumers’ impulsive purchase intention (Wen and Ye, 2014).

**TABLE 5:** Curiosity as a test of the mediating model.

	Curiosity		Impulse purchase intention		Impulse purchase intention	
	β	*T*	β	*T*	β	*T*
Gender	0.080	0.944	0.112	1.348	0.086	1.095
Age	–0.021	–0.336	–0.070	–1.152	–0.063	–1.099
Income	0.092	2.079*	0.076	1.750	0.046	1.119
Perceived uncertainty	0.354	7.378**	0.216	4.583**	0.103	2.026*
Curiosity					0.319	4.708**
*R* ^2^	0.247		0.125		0.217	
*F*	15.409		6.686		10.383	

*P < 0.05, **P < 0.01.

The bootstrap method was used to test the significance of the mediating effect. As shown in Table 6, the 95% confidence intervals of both direct and indirect paths did not contain 0, indicating that curiosity plays a significant mediating role between perceived uncertainty and impulsive purchase intention. The direct effect of perceived uncertainty on impulse purchase intention was 0.103, accounting for 47.69% of the total effect. The indirect effect of perceived uncertainty on impulse purchase intention through curiosity was 0.113, accounting for 52.31% of the total effect.

**TABLE 6:** Curiosity as a mediator in bootstrap analysis.

Path of influence	Effect	SE	BootLLCI	BootULCI	Percentage in total effect (%)
Indirect effect	0.113	0.028	0.062	0.170	52.31
Direct effect	0.103	0.046	0.008	0.192	47.69
Total effect	0.216	0.047	0.117	0.299	–

### Moderating effect test

The process plug-in in SPSS25.0 was used to analyze the moderating effect. Taking perceived uncertainty as the independent variable, impulse purchase intention as the dependent variable, perceived luck as the moderating variable, and controlling the influential factors of gender, age and income, we used Model1 in the macro program compiled by Hayes to test the moderating effect. It can be seen from Table 7 that the interaction term of perceived uncertainty and perceived luck significantly positively predicts impulsive purchase intention (β = 0.181, *t* = 3.244, *P* < 0.01), indicating that perceived luck positively moderates the relationship between perceived uncertainty and impulsive purchase intention, meaning hypothesis H3 was supported.

**TABLE 7:** A moderating model test of perceived luck.

	Impulse purchase intention		
	Effect	SE	*T*
Gender	0.047	0.066	0.713
Age	–0.060	0.048	–1.249
Income	0.039	0.034	1.148
Perceived uncertainty	0.169	0.042	3.996***
Perceived luck	0.699	0.069	10.197***
Perceived uncertainty × Perceived luck	0.181	0.056	3.244**
*R* ^2^	0.462		
*F*	26.594		

**P < 0.01, ***P < 0.001.

**FIGURE 2:**
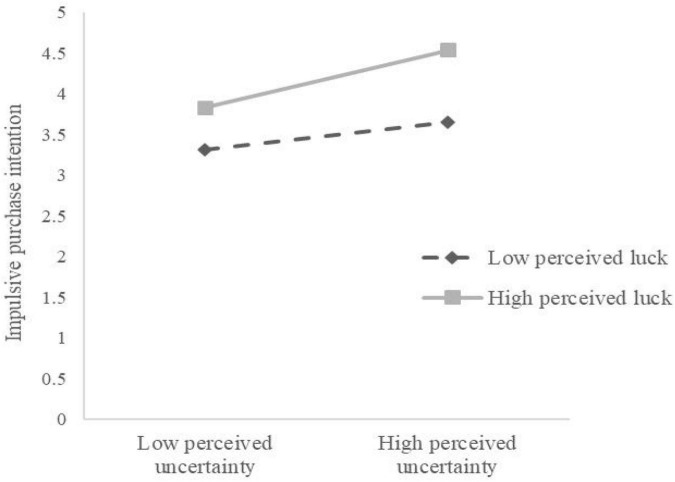
Moderating effect of perceived luck on perceived uncertainty and impulsive purchase intention.

The simple slope analysis in Table 8 shows that, at a low perceived luck level (M-1 SD), perceived uncertainty has no significant predictive effect on impulse purchase intention (β = 0.069, *t* = 1.687, *P* > 0.05). However, at a high perceived luck level (M+1 SD), perceived uncertainty significantly positively predicts impulsive purchase intention (β = 0.270, *t* = 4.347, *P* < 0.001). In other words, with the improvement of consumers’ perceived luck, the positive predictive effect of perceived uncertainty on impulsive purchase intention gradually increases. The moderating effect diagram in Figure 2 illustrates this conclusion more intuitively.

**TABLE 8:** Direct effects of perceived luck at different levels.

	Perceived luck (M ± 1 SD)	Effect	SE	LLCI	ULCI
Total effect	−0.556 (M-1 SD)	0.069	0.041	−0.012	0.149
	0 (M)	0.169	0.042	0.086	0.253
	0.556 (M+1 SD)	0.270	0.062	0.147	0.392

Judging from the current situation of the blind box consumption market, consumers who purchase blind boxes are mainly aged between 18 and 35, and most of them are female (Wang and Bi, 2021). Besides, the sample data collected in this study also that females are more than males, and the sample distribution is basically consistent with the actual consumption data. Based on the gender differences and sample distribution differences in blind box consumption, this paper further explores the role of gender between uncertain rewards and impulsive purchase intention. First, we analyzed the gender differences in impulsive purchase intention using an independent sample t-test. As shown in Table 9, we can see that the gender differences are not significant (*p* > 0.05). Furthermore, we took gender as a moderating variable to test its moderating effect in each influence path. The results show that the interaction term of perceived uncertainty and gender has no significant effect on impulsive purchase intention (β = −0.0001, *t* = −0.001, *p* = 0.999), the interaction term of perceived uncertainty and gender has no significant effect on curiosity (β = −0.078, *t* = −0.743, *p* = 0.459), and the interaction term of curiosity and gender has no significant effect on impulsive purchase intention (β = 0.039, *t* = 0.289, *p* = 0.773). It can therefore be concluded that this research model is not affected by gender.

**TABLE 9:** The result of *t*-test.

	Gender (Mean ± standard deviation)		*T*	*P*
	Male (55)	Female (138)		
Impulse purchase intention	3.47 ± 0.52	3.55 ± 0.55	−0.913	0.362

### Robustness test

Two robustness tests were carried out under the framework of the original theoretical model. Firstly, the independent variable of perceived uncertainty was virtually assigned. That is, according to the median of the selected data, the data of perceived uncertainty was divided into high and low levels for virtual assignment (low-level effect inference value is 0, high-level effect inference value is 1). The virtualized independent variable was replaced with the original variable, and regression analysis was repeated to verify the validity of the theoretical model and assumptions (Zhu and Dong, 2021). Moreover, to further verify the robustness of the conclusion, we collected sample data from 150 consumers who had never experienced purchasing a blind box. Similarly, a regression analysis was performed on the sample data. The results are shown in test 1 and test 2 in Table 10. It can be seen that only the regression coefficient value has changed slightly, but the significance of each path did not change. Therefore, the theoretical model and empirical analysis results of this study have strong reliability and stability.

**TABLE 10:** The robustness test of the mediating effect of curiosity.

	Result variable	Predictive variable	*R* ^2^	*F*	β	*T*
Test 1	Curiosity		0.058	2.866		
		Gender			0.530	0.554
		Age			–0.034	–0.492
		Income			0.124	2.520*
		Perceived uncertainty			0.206	2.389*
	Impulse purchase intention		0.048	2.350		
		Gender			0.102	1.167
		Age			–0.077	–1.221
		Income			0.097	2.146*
		Perceived uncertainty			0.160	2.030*
	Impulse purchase intention		0.206	9.680		
		Gender			0.082	1.027
		Age			–0.065	–1.114
		Income			0.050	1.203
		Perceived uncertainty			0.084	1.137*
		Curiosity			0.373	6.098***
Test 2	Curiosity		0.344	19.017		
		Gender			0.052	0.461
		Age			–0.118	–1.462
		Income			0.082	1.345
		Perceived uncertainty			0.578	8.599***
	Impulse purchase intention		0.297	15.334		
		Gender			0.016	0.151
		Age			–0.007	–0.085
		Income			0.041	0.703
		Perceived uncertainty			0.495	7.776***
	Impulse purchase intention		0.363	16.381		
		Gender			0.011	0.104
		Age			0.028	0.375
		Income			0.017	0.305
		Perceived uncertainty			0.328	4.390***
		Curiosity			0.289	3.841***

*P < 0.05, ***P < 0.001.

## Conclusion and Implications

### Research conclusion

Nowadays, the blind box has become a trending toy and a new trend of social interest favored by young consumers. However, the research on this new phenomenon of blind box marketing in academia is still in its infancy, and most of the literature on the blind box is qualitative analysis. By means of quantitative research methods, this paper deeply explores the internal mechanism of the impact of perceived uncertainty on consumers’ impulsive purchase intention for blind boxes. Meanwhile, we introduce the variables of curiosity and perceived luck to examine the cognitive and emotional motivation behind consumers’ purchase of blind boxes.

The results of this empirical research show that perceived uncertainty has a significant predictive effect on consumers’ impulsive purchase intention, which is consistent with the views put forward by Laran and Tsiros (2013), and Yang et al. (2017). In addition, we explored the mediating effect of curiosity. The result shows that perceived uncertainty has a positive impact on curiosity, further corroborating the findings of Loewenstein (1994), and van Lieshout et al. (2021). Curiosity also has a positive impact on impulsive purchase intention, supporting the views of Hill et al. (2016), and Gruber and Ranganath (2019). In other words, perceived uncertainty can directly affect impulsive purchase intention, and can also indirectly predict impulsive purchase intention through curiosity. We also explored the moderating role of perceived luck. The result shows that perceived luck moderates the relationship between perceived uncertainty and impulsive purchase intention. Specifically, the direct predictive effect of perceived uncertainty on impulsive purchase intention is more significant for individuals with high perceived luck compared to individuals with low perceived luck.

### Theoretical contributions

The theoretical contributions of this study are twofold:

(1) In reviewing the existing literature on the research field of blind box consumption, many scholars have conducted detailed analyses of the blind box economic market and the blind box marketing model (Yang and Li, 2021; Liu, 2022), but there is a relative lack of research on the consumer psychological mechanism behind the blind box consumption, and only some scholars have explored the impact of social support, emotional motivation and customer experience on purchase decisions of blind boxes (Yan and Wu, 2021; Wang et al., 2022). Different from existing studies, this paper takes the uncertain reward of a blind box as a new perspective to explore the internal relationship between perceived uncertainty and impulsive purchase intention. Although the research on uncertain promotion in the field of marketing has achieved rich results, the academic community has not revealed the impact of uncertain rewards in the new phenomenon of blind box marketing on subsequent consumption decisions. Therefore, this study takes the perceived uncertainty of the blind box as the antecedent variable to explore its internal impact on consumers’ impulsive purchase intentions, which provides an expansion direction for the follow-up studies of perceived uncertainty theory and blind box marketing theory and provides a reference for enterprises to carry out uncertain reward marketing strategies in the future.
(2) The selection of mediating variables and moderating variables: preceding researchers have widely discussed the effectiveness of uncertain rewards in promotional activities. Existing studies have pointed out that consumers sometimes prefer uncertain rewards. Uncertain rewards have more significant incentive components than determined rewards, which can stimulate consumers’ positive emotions and promote sales growth. The positive emotions brought by uncertainty may include positive experiences such as surprise, excitement, and imagination (Lee and Qiu, 2009; Laran and Tsiros, 2013; Zhang et al., 2021). The existing literature mainly studies the effect of uncertain promotion from the perspective of these three kinds of emotions. To further explore whether consumer attitudes and behaviors caused by uncertain rewards are also affected by other psychological variables, this study introduces curiosity as an intermediary variable. Although some scholars have previously explored the impact of curiosity on indulgent choice and purchase motivation (Hill et al., 2016; Wang and Huang, 2018), our paper is one of the first that takes curiosity as an intermediary variable to explore its role in the relationship between perceived uncertainty and impulsive purchase intention. In addition, this study also introduces perceived luck as a moderating variable. At present, research on luck mostly takes perceived luck as an antecedent variable to explore its impact on motivation, such as indulgent consumption, gambling frequency, etc. (Zhou, 2018; Jiang et al., 2021). This study verifies the moderating effect of perceived luck on perceived uncertainty and impulsive purchase intention. To a certain extent, this study enriches the related research in the uncertain reward and consumer behavior field.

### Management implications

The results of the study indicate that in the process of uncertain consumption experience of blind boxes, individuals’ curiosity and perceived luck will affect their purchase motivation. As informed by consumer psychology, future enterprises can use the following marketing strategies to enhance marketing performance when they launch a new product.

(1) Keep the mystery of merchandise. In general, people always have the desire to explore unknown things. Providing all the information on commodities will greatly reduce consumers’ desire for further participation. Therefore, marketers can use the information gap to stimulate people’s curiosity for word-of-mouth marketing. For example, managers can release a small amount of information before the launch of a new product. Then they release a little more information at specific intervals to gradually induce consumers’ level of curiosity and maintain their interest in the product.
(2) Add lucky elements to the marketing. According to the above research, individuals with a high perception of luck will optimistically estimate the probability of a good outcome and increase the likelihood of action in uncertain situations. In view of this consumer personality trait, enterprises can integrate content with lucky elements into products to stimulate consumers’ desires to purchase. For example, different lucky seats are set up in restaurants every day, and consumers who sit at these designated seats can enjoy free orders or discounts.

### Limitations and further research

Based on the relevant content of existing uncertain rewards, this study explores the internal influence of perceived uncertainty on consumers’ impulsive purchase intentions. However, this study needs to be further developed and improved, the following outlines specific limitations:

(1) Most of the respondents in this study are young consumer groups aged 19–30, so the conclusion of this study is affected by an incomplete sample range to some extent, and it is difficult to ensure good external validity. Therefore, future research must draw a more comprehensive and extensive sample of participants.
(2) The study did not give full play to the role of basic population information data. Subsequent studies can further discuss whether the factors of age, income, etc. play important roles in the relationship between uncertain reward and purchase intention.
(3) The study only explored the impact of perceived uncertainty on impulsive purchase intention. In the future, the impact of the resolution of uncertain utility on subsequent consumers’ attitudes and behaviors (such as repeated purchase intention or addictive consumption) can be further examined. In addition, the presence of uncertainty means the existence of risk, and we can also further study the effect of individual risk preference on the influence of uncertain reward on purchase intention.

## Data availability statement

The raw data supporting the conclusions of this article will be made available by the authors, without undue reservation.

## Author contributions

YZ contributed to the conception and design of the study and revised the first draft. HZ conducted statistical analysis and wrote the first draft of the manuscript. JQ contributed to the analysis, interpretation of the data, and further revised the contents of the manuscript. All authors contributed to the article and approved the submitted version.

## References

[B1] Ailawadi K. L. Gedenk K. Langer T. Ma Y. Neslin S. A. (2014). Consumer response to uncertain promotions: an empirical analysis of conditional rebates. *Int. J. Res. Market.* 31 94–106. 10.1016/j.ijresmar.2013.08.002

[B2] Bagozzi R. P. Gopinath M. Nyer P. U. (1999). The role of emotions in marketing. *J. Acad. Market. Sci.* 27 184–206. 10.1177/0092070399272005

[B3] Bar-Hillel M. Budescu D. (1995). The elusive wishful thinking effect. *Think. Reason.* 1 71–103. 10.1080/13546789508256906 18488640

[B4] Becker M. C. Knudsen T. (2005). The role of routines in reducing pervasive uncertainty. *J. Bus. Res.* 58 746–757. 10.1016/j.jbusres.2003.10.003

[B5] Berlyne D. E. (1978). Curiosity and learning. *Motiv. Emot.* 2 97–175. 10.1007/BF00993037

[B6] Brod G. Breitwieser J. (2019). Lighting the wick in the candle of learning: generating a prediction stimulates curiosity. *NPJ Sci. Learn.* 4 1–7. 10.1038/s41539-019-0056-y 31646002 PMC6803639

[B7] Calvo M. G. Dolores Castillo M. (2001). Selective interpretation in anxiety: uncertainty for threatening events. *Cogn. Emot.* 15 299–320. 10.1080/02699930126040

[B8] Carver C. S. Scheier M. F. Segerstrom S. C. (2010). Optimism. *Clin. Psychol. Rev.* 30 879–889. 10.1016/j.cpr.2010.01.006 20170998 PMC4161121

[B9] Darke P. R. Freedman J. L. (1997a). Lucky events and beliefs in luck: paradoxical effects on confidence and risk-taking. *Personal. Soc. Psychol. Bull.* 23 378–388. 10.1177/0146167297234004

[B10] Darke P. R. Freedman J. L. (1997b). The belief in good luck scale. *J. Res. Personal.* 31 486–511. 10.1006/jrpe.1997.2197 14650692

[B11] Day L. Maltby J. (2005). “With good luck”: belief in good luck and cognitive planning. *Personal. Ind. Differ.* 39 1217–1226. 10.1016/j.paid.2005.04.011

[B12] Goldsmith K. Amir O. (2010). Can uncertainty improve promotions? *J. Market. Res.* 47 1070–1077. 10.1509/jmkr.47.6.1070 11670861

[B13] Gruber M. J. Ranganath C. (2019). How curiosity enhances hippocampus-dependent memory: the prediction, appraisal, curiosity, and exploration (PACE) framework. *Trends Cogn. Sci.* 23 1014–1025. 10.1016/j.tics.2019.10.003 31706791 PMC6891259

[B14] Guo Z. Chen J. Liu S. Li Y. Sun B. Gao Z. (2013). Brain areas activated by uncertain reward-based decision-making in healthy volunteers. *Neural Regenerat. Res.* 8 3344–3352. 10.3969/j.issn.1673-5374.2013.35.009 PMC4145940 25206656

[B15] Hill K. M. Fombelle P. W. Sirianni N. J. (2016). Shopping under the influence of curiosity: how retailers use mystery to drive purchase motivation. *J. Bus. Res.* 69 1028–1034. 10.1016/j.jbusres.2015.08.015

[B16] Hock S. J. Bagchi R. Anderson T. M. (2020). Promotional games increase consumer conversion rates and spending. *J. Consum. Res.* 47 79–99. 10.1093/jcr/ucz043

[B17] Holbrook M. B. (1986). “Emotion in the consumption experience: toward a new model of the human consumer,” in *The Role of Affect in Consumer Behavior: Emerging Theories and Applications*, Vol. 6, (Lexington, MA: D.C. Heath), 17–52.

[B18] Huang S. H. Xiao J. C. Jin Y. N. (2020). Study on the influencing factors of consumers’ continuous buying intention on social e-commerce platform based on SOR theory. *Soft Sci.* 34 115–121.

[B19] Jezzini A. Bromberg-Martin E. S. Trambaiolli L. R. Haber S. N. Monosov I. E. (2021). A prefrontal network integrates preferences for advance information about uncertain rewards and punishments. *Neuron* 109 2339–2352. 10.1016/j.neuron.2021.05.013 34118190 PMC8298287

[B20] Jiang D. Zhu H. Wang X. Xiao C. Q. (2021). The permissive effect of luck: The influence of luck perception on indulgent consumption. Collect. *Essays Finance Econ.* 273, 90–100.

[B21] Kaltcheva V. D. Weitz B. A. (2006). When should a retailer create an exciting store environment? *J. Market.* 70 107–118. 10.1509/jmkg.70.1.107.qxd 11670861

[B22] Kashdan T. B. Gallagher M. W. Silvia P. J. Winterstein B. P. Breen W. E. Terhar D. (2009). The curiosity and exploration inventory-II: development, factor structure, and psychometrics. *J. Res. Personal.* 43 987–998. 10.1016/j.jrp.2009.04.011 20160913 PMC2770180

[B23] Laran J. Tsiros M. (2013). An investigation of the effectiveness of uncertainty in marketing promotions involving free gifts. *J. Market.* 77 112–123. 10.1509/jm.11.0255 11670861

[B24] Lee Y. H. Qiu C. (2009). When uncertainty brings pleasure: the role of prospect imageability and mental imagery. *J. Consum. Res.* 36 624–633. 10.1086/599766

[B25] Liu S. L. (2022). People in a box: blind box consumption landscape and its formation mechanism of Z generation. *China Youth Study* 2 78–84.

[B26] Loewenstein G. (1994). The psychology of curiosity: a review and reinterpretation. *Psychol. Bull.* 116 75–98. 10.1037/0033-2909.116.1.75

[B27] Ma Q. G. (2002). Several key problems faced by management science research in China. *Manage. World.* 18, 105–115.

[B28] Mehrabian A. Russell J. A. (1974). *An Approach to Environmental Psychology.* Cambridge, MA: MIT Press.

[B29] Menon S. Soman D. (2002). Managing the power of curiosity for effective web advertising strategies. *J. Adv.* 31 1–14. 10.1080/00913367.2002.10673672

[B30] Pizzagalli D. A. Iosifescu D. Hallett L. A. Ratner K. G. Fava M. (2008). Reduced hedonic capacity in major depressive disorder: evidence from a probabilistic reward task. *J. Psychiatr. Res.* 43 76–87. 10.1016/j.jpsychires.2008.03.001 18433774 PMC2637997

[B31] Quintal V. A. Lee J. A. Soutar G. N. (2010). Risk, uncertainty and the theory of planned behavior: a tourism example. *Tour. Manage.* 31 797–805. 10.1016/j.tourman.2009.08.006

[B32] Ruan B. Hsee C. K. Lu Z. Y. (2018). The teasing effect: an underappreciated benefit of creating and resolving an uncertainty. *J. Market. Res.* 55 556–570. 10.1509/jmr.15.0346 11670861

[B33] Shen L. Fishbach A. Hsee C. K. (2015). The motivating-uncertainty effect: uncertainty increases resource investment in the process of reward pursuit. *J. Consum. Res.* 41 1301–1315. 10.1086/679418

[B34] Shi H. Chen R. Xu X. (2021). How reward uncertainty influences subsequent donations: the role of mental accounting. *J. Bus. Res.* 132 383–391. 10.1016/j.jbusres.2021.04.040

[B35] Shou Z. G. Teng H. X. Zheng W. H. Peng Z. J. (2021). How to take advantage of uncertainty: The impact of gambled games on service recovery. *Nankai Bus. Rev.* 25, 1–20.

[B36] Singh A. Manjaly J. A. (2021). The effect of information gap and uncertainty on curiosity and its resolution. *J. Cogn. Psychol.* 33 403–423. 10.1080/20445911.2021.1908311

[B37] Taylor S. E. Brown J. D. (1988). Illusion and well-being: a social psychological perspective on mental health. *Psychol. Bull.* 103 193–210. 10.1037/0033-2909.103.2.193 3283814

[B38] Thomas M. R. Mathew J. (2018). Online merchandising cues influencing the purchase intention of generation Z mediated by emotions using-SOR framework. *Asian J. Manage.* 9 175–182. 10.5958/2321-5763.2018.00027.6

[B39] van Lieshout L. L. de Lange F. P. Cools R. (2021). Uncertainty increases curiosity, but decreases happiness. *Sci. Rep.* 11 1–10. 10.1038/s41598-021-93464-6 34234250 PMC8263743

[B40] Von Neumann J. Morgenstern O. (2007). *Theory of Games and Economic Behavior.* Princeton, NJ: Princeton University Press.

[B41] Wang C. Huang Y. (2018). “I want to know the answer! Give me Fish’n’Chips!”: the impact of curiosity on indulgent choice. *J. Consum. Res.* 44 1052–1067. 10.1093/jcr/ucx086

[B42] Wang T. Y. Bi S. M. (2021). Marketing mode of trendy toy “blind box”. *Mod. Bus.* 612, 15–17.

[B43] Wang W. J. Meng Y. Q. Xu M. Y. (2022). Analysis of the driving mechanism of social support and emotional motivation on blind box consumption. *J. Commercial Econ.* 13 62–67.

[B44] Ward J. C. Hill R. P. (1991). Designing effective promotional games: opportunities and problems. *J. Advert.* 20 69–81. 10.1080/00913367.1991.10673348

[B45] Weiss D. J. (1970). Factor analysis and counseling research. *J. Counsel. Psychol.* 17 477–485. 10.1037/h0029894

[B46] Wen Z. L. Ye B. J. (2014). Analyses of mediating effects: the development of methods and models. *Adv. Psychol. Sci.* 22 731–745. 10.3724/SP.J.1042.2014.00731

[B47] Wohl M. J. Enzle M. E. (2003). The effects of near wins and near losses on self-perceived personal luck and subsequent gambling behavior. *J. Exp. Soc. Psychol.* 39 184–191. 10.1016/S0022-1031(02)00525-5

[B48] Wood M. (2005). Discretionary unplanned buying in consumer society. *J. Consum. Behav. Int. Res. Rev.* 4 268–281. 10.1002/cb.14

[B49] Yan X. Wu J. F. (2021). The effect of blind box customer experience on repeated purchase intention. *China Bus. Market* 35 85–95.

[B50] Yang F. X. Li A. Q. (2021). Adult fairy tales: a study of urban youth’s blind box consumption. *J. Jiangxi Normal Univ. Philos. Soc. Sci. Edn.* 54 65–74.

[B51] Yang Y. Gu Y. Galak J. (2017). When it could have been worse, it gets better: how favorable uncertainty resolution slows hedonic adaptation. *J. Consum. Res.* 43 747–768. 10.1093/jcr/ucw052

[B52] Yarritu I. Matute H. Vadillo M. A. (2014). Illusion of control: the role of personal involvement. *Exp. Psychol.* 61 38–47. 10.1027/1618-3169/a000225 23948387 PMC4013923

[B53] Zhang G. Liu Z. Y. (2010). Effects of influential factors on consumer perceptions of uncertainty for online shopping. *Nankai Bus. Rev.* 13, 99–106.

[B54] Zhang J. M. Kong W. Z. (2021). The Influence of negative online word-of-mouth on consumers’ impulsive purchasing intentions: the mediating effect of negative emotions. *Manage. Rev.* 33 144–156.

[B55] Zhang Y. J. Lee Y. Kim S. H. (2017). Effect of motivation type and reward uncertainty on consumers’ marketing promotion participation. *Asia Market. J.* 19 45–74. 10.15830/amj.2017.19.3.45

[B56] Zhang Y. X. Wu M. Z. Ma Q. H. (2021). Will free gifts please customers: view under benefit congruency framework. *J. Northeastern Univ. Soc.Sci.* 23 38–47.

[B57] Zhou J. (2018). The relationship between gambling socialization, beliefs in luck and college students’ gambling frequency. *Chine. J. Spec. Educ.* 216, 78–83.

[B58] Zhu L. Li H. Wang F. K. He W. Tian Z. (2020). How online reviews affect purchase intention: a new model based on the stimulus-organism-response (SOR) framework. *Aslib J. Inform. Manage.* 72 463–488. 10.1108/AJIM-11-2019-0308

[B59] Zhu X. M. Dong Z. (2021). The influence of lean startup on entrepreneurial bricolage. *Stud. Sci. Sci.* 39 295–302.

